# Experiences of autistic and non-autistic individuals participating in a corporate internship scheme

**DOI:** 10.1177/13623613211025115

**Published:** 2021-06-19

**Authors:** Anna Remington, Brett Heasman, Anna Melissa Romualdez, Elizabeth Pellicano

**Affiliations:** 1 University College London, UK; 2 Macquarie University, Australia; 3 York St John University, UK

**Keywords:** autism, employment, internship, outcomes, work

## Abstract

Autistic people have significant challenges in obtaining and maintaining employment yet there remains a shortage of research in this area, especially research directly comparing the experiences of autistic individuals to their non-autistic colleagues in the same organisation. The present study examined the experiences of autistic and non-autistic interns, and their managers, taking part in a corporate internship scheme. Data were gathered via semi-structured interviews and online questionnaires prior to, and following, the internship. Many commonalties were identified, with both groups of interns and managers sharing positive journeys through the internship. Specific issues raised by autistic interns centred around impact of prior employment experiences, mental health and communication. Compared to managers of non-autistic interns, managers of autistic interns reported having a greater range of pre-internship concerns, including concerns about providing the right level of support, communicating successfully and being equitable in treatment of all employees. Structured delegation of tasks and flexible communication were successful strategies used by managers to support autistic interns; clear communication and more consistent support were perceived to benefit both intern groups. The findings highlight specific challenges experienced by autistic individuals in the workplace and suggest effective ways to ensure that autistic interns succeed alongside non-autistic peers.

**Lay abstract:**

Autistic people can find it difficult to find and keep a job, and fewer autistic people are employed compared with people from other disability groups. There is not enough research in this area, especially research that directly compares the experiences of autistic and non-autistic colleagues starting in an organisation at the same time. Our study looked at the experiences of autistic and non-autistic people taking part in an internship at Deutsche Bank, UK. We spoke to the interns before the internship began, and again once it had finished. We also asked the interns’ hiring managers about their experiences of the internship. We used interviews and online questionnaires to find out people’s views. Before the programme began, managers of autistic interns were more worried about the internship than managers of the non-autistic interns. They were worried about providing the right level of support, communicating successfully and treating all their employees fairly. At the end of the internship, everyone felt that the internship was a success. Managers of autistic interns explained how the experience had made them better managers. Both groups of interns and said that they benefitted from clear communication and would have likes more support. Managers of autistic interns spoke about dividing tasks up into smaller chunks and being flexible in their communication were helpful when working with the autistic interns. More work is needed to make sure that autistic interns are integrated alongside non-autistic peers. One way to make this happen might be to create guides for managers.

## Introduction

Many autistic people are willing and able to work (Baldwin et al., 2014). Yet, across the world, autistic people consistently experience poor employment prospects (Billstedt et al., 2005; Buescher et al., 2014; Holwerda et al., 2013). In the United Kingdom, autistic adults are less likely to be in full-time employment, or indeed any form of employment, than people belonging to any other disability group (National Autistic Society, 2016). Consequently, there has been increased statutory guidance in the United Kingdom aimed at creating more jobs and improving access for autistic people into work (Connolly et al., 2016; Department of Health, 2014). Indeed, research has drawn attention to the particular strengths that autistic people bring to the workplace, including attention to detail, logical reasoning, passionate interests and loyalty (Anderson et al., 2021; Lorenz & Heinitz, 2014). Of most relevance to the present study, Remington and Pellicano (2019) reported that autistic graduates participating in a bank internship were able to contribute successfully to their teams and they, like their co-workers, found the overall process to be hugely positive.

Despite the clear contributions that autistic people can make in the workplace, much of the existing work on employment for autistic individuals is rooted within a medical model and is therefore overwhelmingly focussed on individual impairments and challenges (Scott et al., 2018). Frequently reported obstacles for autistic employees include difficulties around interpersonal communication, relationship management, learning and applying knowledge, task management and mental health management, which align with the social and cognitive challenges associated with being autistic (Barnhill, 2007; Chen et al., 2015; Hendricks, 2010; Lorenz et al., 2016). Studies that have explored the qualitative experiences of autistic people in employment report similar barriers. For example, Griffith et al. (2012) examined the experiences of autistic adults and found that anxiety and stress around communication could make work overwhelming and lower an individual’s sense of self-worth (Griffith et al., 2012).

Research on how best to support autistic employees to obtain and maintain employment needs to be furthered in a number of areas. First, much of our understanding about the barriers autistic people face has been developed on the basis of sampling autistic people only, with systematic reviews highlighting a lack of adequate non-autistic comparisons as a significant limitation of existing research (Hedley et al., 2017; Scott et al., 2018). Indeed, non-autistic people also experience challenges associated with anxieties around entering employment, embedding themselves within a new company culture and speaking up about their concerns (Frenette, 2013; Noort et al., 2019). Identifying barriers that are specific to autistic employees can facilitate much needed autism-focussed workplace support (Anderson et al., 2021; Richards, 2012).

Second, a lack of multi-informant studies undermines our understanding of the full context within which the autistic employees work. For example, the perspectives of hiring managers and co-workers are integral to addressing issues such as stigma, which has been identified as a major barrier to disabled people gaining employment and an issue that impacts workplace performance (Mclaughlin et al., 2004).

Third, a limitation of previous research is that it has often focussed on one point in time, which means that aspects of employment that take longer to navigate are not well understood. This issue is particularly pertinent given that key challenges experienced by autistic people in the workplace often centre on communication, which is a process that takes time to develop within an organisation (Suppiah & Sandhu, 2011). Furthermore, similar challenges include learning tacit skills that extend beyond the explicit requirements of the job role (Gal et al., 2015) and learning new processes such as how to optimise physical environments towards the sensory needs of the individual (Hillier et al., 2007). Here, we report a comparison of employee experiences across different time-points, an approach that is integral to understanding how autistic people and their employers face and overcome challenges as they go through the process of starting a new role within an organisation.

### The current study

The present study builds on previous research examining a 3-month graduate internship programme at Deutsche Bank (DB), in London, UK (Remington & Pellicano, 2019). Workplace internships like this one are periods of supervised work within a company to enable prospective candidates to gain valuable employment experience, especially if they have been disadvantaged within the education system prior to seeking a job (Shattuck et al., 2012; Wehman et al., 2014). For autistic people, work placements can be advantageous to those who require time to become familiar with a new set of practices and routines specific to organisations or who may require longer processing time for new information. They offer the chance to demonstrate skills that may not be so easy for autistic people to articulate in the pressures of a face-to-face job interview and therefore have the potential to act as a gateway to subsequent longer-term contracts.

Here, we compared the experiences of autistic *and* non-autistic interns involved in DB’s internship programme, using a multi-informant, mixed approach. To this end, we conducted semi-structured interviews with interns and their managers both before and after the internship, focusing on pre-internship expectations and post-internship reflections. We examined the expectations and reflections of interns’ managers to compare experiences and understand how strategies for best practice are specifically adapted to support neurodiversity. We also used quantitative measures of workplace performance, well-being and neurodiversity to understand the experiences of the two groups.

## Method

### Internship programme

Deutsche Bank (DB) employs between 9000 and 10,000 people in the United Kingdom alone, with an international presence that spans several continents. Each summer, the bank runs a 3-month internship programme with the goal of introducing young graduates to potential careers. Applicants must be on course to achieve a degree result of 2:1 or higher^1^ and set to graduate the following year. The recruitment process involves an online application form and situational judgement test, a telephone interview and an assessment day. Approximately 180 interns were accepted to the programme in 2018.

In 2016, DB UK partnered with UK autism research charity Autistica to create and implement an autistic graduate internship programme. The current study involved participants who took part in the second and third iterations of the programme, from July to September 2017 and July to September 2018. The hiring managers involved in the internship programme were those who volunteered in response to an email sent within DB asking for posts for potential interns. Prior to the start of the scheme, training run by Ambitious about Autism, a UK-based autism advocacy organisation, was offered to the DB staff members involved. This training was delivered to staff responsible for conducting the interviews with potential candidates, while mandatory workshops were given to those DB staff members who would be line-managing the autistic graduates throughout the programme.

Autistic interns were recruited through adverts placed on job boards, social networks associated with autism-specific organisations and university disability employment services in the United Kingdom. As with the general DB internship, interested candidates for the autistic internship scheme were asked to submit a CV to assess their eligibility, which included the completion of an undergraduate degree within the past 3 years. Unlike the general internship scheme, however, the interview process involved DB emailing questions to candidates rather than require them to take part in face-to-face testing, to increase the accessibility of recruitment. The questions sent to the candidates were specific to the scheme and covered their previous work experience, as well as abstract reasoning skill. Candidates had 1 week to respond. They were then invited for in-depth, face-to-face interviews with trained DB staff.

All candidates who completed the screening process were offered places on the scheme and were placed in various departments across DB’s five offices in Central London, as well as in the Birmingham office. These placements were in areas that included technology, human resources, operations, risk, computer programming and finance. The interns on the autism-specific internship scheme were given the same salary as those on the regular DB internship scheme. Similar to the regular scheme, the autistic interns were also assigned a volunteer buddy who acted as a mentor. In response to the findings from the initial internship scheme (Remington & Pellicano, 2019), which suggested mentors external to the organisation would benefit both interns and managers participating in the autism-specific internship scheme, DB further provided a mentor from an external company specialising in workplace mentoring for autistic people, AS Mentoring. Each autistic intern was therefore assigned a point person from AS Mentoring, with whom they met regularly throughout the internship for check-ins and additional support. While recommendations from our prior research were used by DB staff to modify the autism-specific internship programme, researchers conducting the present study were not involved in implementing the programme, nor played any part in support or training for interns or managers. This allowed all research to be conducted independently and allowed participants to respond freely without concern that certain views would be unwelcome.

### Participants

All interns and hiring managers involved in both the regular and autistic schemes were invited to take part in our research via emails sent by scheme coordinators within the Bank. Fifty-two adults opted to participate, including all 32 members of the autism-specific graduate scheme (n = 16 autistic interns; n = 16 hiring managers) and 20 out of the 180 interns from the general graduate scheme (n = 15 non-autistic interns; n = 5 hiring managers). Of these participants, one autistic intern and one manager of a non-autistic intern were not able to take part in the pre-internship interviews. Four non-autistic interns and three managers of the autistic interns were not able to take part in the post-internship interviews. The interns and hiring managers were from a variety of departments within the bank, with the hiring managers having worked at DB for varying amounts of time (16 months to 26 years) and in various roles (see Table 1 for demographics).

**Table 1.** Participant demographics.

Variable	Autistic intern (n = 15)	Non-autistic intern (n = 15)	Manager of autistic intern (n = 16)	Manager of non-autistic intern (n = 4)
Age (years)	Mean (range)			
	25	20	45	37
	21–36	19–21	36–60	33–41
Gender	n (%)	n (%)	n (%)	n (%)
Male	14 (93)	8 (53)	9 (56)	3 (75)
Female	1 (7)	7 (47)	7 (44)	1 (25)
Number of previous job applications	n (%)	n (%)	N/A	N/A
None	2 (13)	2 (13)		
1–5	1 (7)	5 (33)		
6–10	1 (7)	3 (20)		
11–15	3 (20)	1 (7)		
16+	6 (40)	1 (7)		
Did not answer	2 (13)	3 (20)		
Years worked at DB	N/A	N/A	Mean (range)	Mean (range)
			9 years (6 months–25 years)	12 years (8–16 years)
Other Conditions	n	n	N/A	N/A
Attention deficit	4	1		
Hyperactivity disorder				
Dyslexia	3			
Dyspraxia	3			
Depression	2			
Anxiety	2	1		
Obsessive compulsive disorder	1			
Social Responsiveness Scale – second edition^a^	Mean (SD) (n = 12)	N/A	N/A	N/A
	65 (12.5)			
Waisman Activities of Daily Living Scale^b^	Mean (SD) (n = 13)	Mean (SD) (n = 12)	N/A	N/A
	29.2 (4.6)	32.6 (0.9)		

DB: Deutsche Bank; SD: standard deviation.

a Constantino and Gruber (2012), higher scores indicate greater challenges with social interaction.

b Maenner et al. (2013), higher scores indicate greater independence.

Most interns spoke English as their first language and all had completed or were in the process of completing university degrees in a range of subjects. Among the non-autistic interns, none reported that they were autistic, although one specified an ADHD diagnosis and one reported having an anxiety disorder. Of the 30 autistic and non-autistic interns who participated in the Deutsche Bank graduate internship schemes, 16 had previous work experience in settings that included corporate offices, retail, IT, research, finance and the public sector.

All autistic interns reported an independent clinical diagnosis of autism, with most (n = 9, 60%) having been diagnosed at an early age but six (40%) stating that they had only received their diagnosis within the last 10 years. The Social Responsiveness Scale – Second edition (Constantino & Gruber, 2012) was used to assess the current autistic features of the autistic interns. Of the 12 who completed the SRS-2, five scored within the ‘normal’ range, three were classified as ‘mild’, two were classified as ‘moderate’ and two were classified as ‘severe’. The Waisman Activities of Daily Living Scale (W-ADL; Maenner et al., 2013) was used to assess the level of independence of both groups of interns. Of the 13 autistic interns who completed the W-ADL, nine scored high on independence (range = 30–33 out of a possible highest score of 34) and four scored in the moderate range (scores of 22–26), indicating a need for assistance in some daily living tasks. All 12 non-autistic interns scored within the range indicative of a high level of independence.

### Procedure

At the start of the internship, semi-structured interviews were conducted with 15 autistic interns, 15 interns from the regular DB graduate scheme, 16 hiring managers from the autism-specific internship scheme and 4 managers from the general scheme. These interviews included questions about their expectations, any concerns they had and their hopes for the programme (see Supplementary Materials for full interview questions).

Interviews lasted approximately 30 min and were conducted over the phone or in person according to individual participant preference. In advance of starting the internship, in addition to the interviews, autistic and non-autistic interns completed a number of measures (see Table 2). All participants gave written consent to take part.

**Table 2.** Quantitative measures completed within the study.

Measure	Construct	Scoring^a^	Example item	Reliability	Participant group and time point
The Work Self-Efficacy Inventory (WS-Ei; Raelin, 2010)	Workplace confidence (higher scores reflect greater confidence)	30 items, rated on 5-point scale from ‘not at all’ to ‘completely’ Maximum overall composite score of 5	How confident are you working under pressure?	Cronbach’s α = 0.97	Interns, pre- and post-internship
Adult ADHD Self-Report Scale (ASRS; Kessler et al., 2005)	ADHD symptoms (higher scores reflect a higher level of ADHD symptoms)	6 items rated on 5-point scale from ‘never’ to ‘very often’ Maximum score of 72	How often do you have difficulty getting things in order when you have to do a task that requires organisation?	Cronbach’s α = 0.83	Interns, pre-internship
Patient Health Questionnaire depression module (PHQ-8); Kroenke et al. (2001)	Level of depression (higher scores reflect a higher level of depression)	8 items rated on a 4-point scale ranging from ‘not at all’ to ‘nearly every day’ Maximum score of 24	How often in the past two weeks have you experienced ‘feeling down, depressed, or hopeless?’	Cronbach’s α = 0.89	Interns, pre- and post-internship
General Anxiety Disorder 7 (GAD-7 Spitzer et al., 2006)	Level of anxiety (higher scores reflecting greater severity of anxiety)	7 items rated on a 4-point scale ranging from ‘not at all’ to ‘nearly every day’ Maximum score of 21	How often have you been bothered by certain feelings, e.g. ‘feeling nervous, anxious, or on edge?’	Cronbach’s α = 0.88	Interns, pre- and post-internship
World Health Organization Quality of Life: Brief Version (WHOQOL-BREF; World Health Organization, 1996; Skevington et al., 2004)	Perception of a person’s standing in life (higher scores reflect better quality of life)	26 items on a 0–100 scale according to four dimensions: physical health, psychological well-being, social relationship and environment Maximum score of 100 for each dimension	How satisfied are you with your sleep?	Cronbach’s α ranging from = 0.69 to 0.94 for the four domains	Interns, pre-internship
Work Performance Questionnaire (WPQ, Modified from the Work Performance Evaluation (WPE; Katz et al., 2015)	Employee performance (frequency and independence when carrying out tasks) (higher scores reflect more frequent and more independent performance on the task in question)	31 items rated on 5-point scale for frequency and independence, split into five subscales: (1) presentation (appearance at work), punctuality and responsibility; (2) task comprehension and planning; (3) task performance; (4) dealing with distractions and (5) contact/interaction with colleagues and superiors. Maximum score of 35 for frequency and 35 for independence on each subscale	Please fill out the questionnaire relating to *Frequency* (how often a behaviour occurs) and *Independence* (whether/how much you/the employee needs any help or assistance to complete the task) for each of the behaviours listed below:	Cronbach’s α = 0.98 for interns and 0.95 for managers	Interns and managers, post-internship

WS-Ei: Work Self-Efficacy Inventory; ADHD: attention deficit hyperactivity disorder; ASRS: Adult ADHD Self-Report Scale; PHQ-8: Patient Health Questionnaire; GAD-7: General Anxiety Disorder 7; WHOQOL-BREF: World Health Organization Quality of Life: Brief Version; WPQ: Work Performance Questionnaire; WPE: Work Performance Evaluation.

At the conclusion of the programme, interns and their managers took part in a second semi-structured interview. All questions were developed prior to the start of the study and asked participants about their experiences of the programme, with a particular focus on successes, challenges and support offered to them during the process (see Supplementary Materials). In addition, the WS-Ei, PHQ-8 and GAD-7 were again completed by the interns. Managers and interns also completed the *Work Performance Questionnaire* (WPQ) modified from the Work Performance Evaluation (WPE; Katz et al., 2015). Ethics approval for this study was granted by the UCL Institute of Education Ethics Committee.

### Community involvement

The design of the study was informed and guided by an autistic intern who had previously participated in the internship scheme.

### Data analysis

The quantitative data were analysed using the Statistical Package for the Social Sciences (IBM SPSS Statistics 26). Non-parametric tests (Mann–Whitney U and Wilcoxon Signed Rank) were used due to the small sample sizes. To avoid Type 1 errors that may arise due to the multiple group comparisons conducted, results were only considered significant if they reached a p value of at least 0.01. Effect sizes were calculated using r_equivalent_ (Rosenthal & Rubin, 2003), a simple effect size indicator that can be used with non-parametric tests. Not all participants completed interviews and questionnaires at both time points, therefore participant numbers are included for each measure (see Table 3).

**Table 3.** Pre- and post-internship measures.

Variable	Autistic interns		Non-autistic interns	
	Pre-internship	Post-internship	Pre-internship	Post-internship
World Health Organization Quality of Life (Brief Version) (WHOQOL-BREF)	Mean (SD) n = 9		Mean (SD) n = 11	
Physical health	64.0 (22.2)^a^	N/A	90.5 (6.6)^a^	N/A
Psychological well-being	52.9 (25.7)^a^	N/A	84.1 (16.1)^a^	N/A
Social interaction	61.9 (38.3)	N/A	80.2 (17.6)	N/A
Environment	63.2 (28.4)^a^	N/A	93.3 (7.6)^a^	N/A
Adult ADHD Self-Report Scale (ASRS)	Mean (SD) n = 9		Mean (SD) n = 11	
	10 (5.0)	N/A	4.8 (4.3)	N/A
Personal Health Questionnaire Depression Scale (PHQ-8)	Mean (SD) n = 9	Mean (SD) n = 11	Mean (SD) n = 11	Mean (SD) n = 8
	7.4 (6.6)	9.7 (7.6)	1.6 (1.5)	5.0 (6.7)
Generalised Anxiety Disorder Assessment (GAD-7)	Mean (SD) n = 9	Mean (SD) n = 11	Mean (SD) n = 12	Mean (SD) n = 8
	5.7 (5.3)	8.4 (8.3)	2.5 (2.6)	4.4 (5.8)
Work Self-Efficacy Inventory (WSE-i)	Mean (SD) n = 9	Mean (SD) n = 11	Mean (SD) n = 11	Mean (SD) n = 8
	3.1 (0.5)^a^	3.5 (0.6)	4.4 (0.3)^a^	4.0 (0.6)

WHOQOL-BREF: World Health Organization Quality of Life: Brief Version; SD: standard deviation; ADHD: Attention deficit hyperactivity disorder; ASRS: Adult ADHD Self-Report Scale; PHQ-8: Patient Health Questionnaire; GAD-7: General Anxiety Disorder 7; WSE-i: Work Self-Efficacy Inventory.

a Significant difference between the autistic and non-autistic groups.

Audio recordings of interviews were transcribed verbatim and were entered into NVivo 12 Pro (2018). We used Braun and Clarke’s process of reflexive thematic analysis (Braun & Clarke, 2019). Our epistemological stance fits within a critical realist framework, which acknowledges that we all have subjective experiences (the empirical), that an objective reality exists outside of our experience (the actual) and that causal mechanisms lie between and within these domains (the real; Willig, 2013). We used inductive methods to identify themes, by identifying patterns in the data without integrating them within pre-existing codes or preconceptions.

Qualitative data within a longitudinal approach require analysis in two directions: cross-sectionally (to examine group differences) and longitudinally (to examine progression over time; Holland, 2007; Thomson & Holland, 2003). As such, the data in the present study were grouped into four sets for analysis, with interns (autistic vs non-autistic) and managers (of autistic vs non-autistic interns) coded separately for each time point, and remained separate during the identification of themes. This procedure provided a view of cross-sectional group differences. Two researchers led the coding of the transcripts: B.H. read all transcripts to familiarise himself with the data and developed initial codes in discussion with M.R. B.H. then coded the manager data, while M.R. coded the intern data (using the initial codes). B.H. and M.R. met multiple times to discuss and refine codes, as required. The pre- and post-internship data were then merged in order to allow longitudinal themes to be extracted. Through an iterative process of refinement, the researchers met several times to review progress and decide on the themes that best fit the data. All authors approached the analysis and discussions from the perspective of researchers who do not identify as autistic, although some authors do identify as neurodivergent.

In the interest of confidentiality, quotations are reported using pseudonymised IDs assigned to participants. Given the few female participants, all references to participants are made using male pronouns to preserve their anonymity.

## Results

### Quantitative results

At the beginning of the programme, the non-autistic interns scored significantly higher than the autistic interns for three out of four dimensions of the WHOQOL-BREF: physical health (p = 0.003, r_equivalent_ = −0.65), psychological well-being (p = 0.006, r_equivalent_ = −0.61) and environment (p = 0.002, r_equivalent_ = −0.68). There was no significant difference in the satisfaction reported for social interaction (p = 0.37, r_equivalent_ = −0.21). The non-autistic interns also scored significantly higher on the WS-Ei (p < 0.001, r_equivalent_ = −0.82), revealing greater confidence in their work-related abilities compared to their autistic peers. There was no significant group difference, however, on level of anxiety (as measured by GAD-7 scores, p = 0.095, r_equivalent_ = 0.37), and the higher levels of ADHD traits (as measured by ASRS scores, p = 0.025, r_equivalent_ = 0.50) and depression (as measured by PHQ-8 scores, p = 0.046, r_equivalent_ = 0.46) reported by the autistic interns were not significant after correcting for multiple comparisons. Post-internship there were no significant differences between the scores of autistic and non-autistic interns on measures of depression (p = 0.129, r_equivalent_ = 0.35) or anxiety (p = 0.351, r_equivalent_ = 0.22). The difference in work self-efficacy (as measured by WS-Ei scores, p = .020, r_equivalent_ = −0.53) was not significant after correcting for multiple comparisons.

There were no significant changes over time on the PHQ-8, GAD-7 or WS-Ei scores (p = 0.310, r_equivalent_ = 0.27) for the autistic or non-autistic interns (all p values > 0.25). Table 3 shows the breakdown of scores for both groups on each of these measures.

With respect to work performance (WPQ scores), the non-autistic interns rated themselves significantly higher on their ability to engage in task comprehension and planning (p = 0.004) and felt they could more frequently deal with distractions successfully (p = 0.001). There were no other significant group differences in WPQ self-ratings. The managers’ scores revealed that they felt non-autistic interns could more frequently comprehend and plan tasks than the autistic interns (p = 0.006) and that the non-autistic interns showed a higher level of independence when dealing with distractions (p = 0.006). There were no other significant differences in WPQ ratings of interns by managers on the autism-specific and general internship schemes (see Supplementary Materials for breakdown of scores on each domain).

### Qualitative results

#### Interns’ experiences through the programme

**Figure 1.**
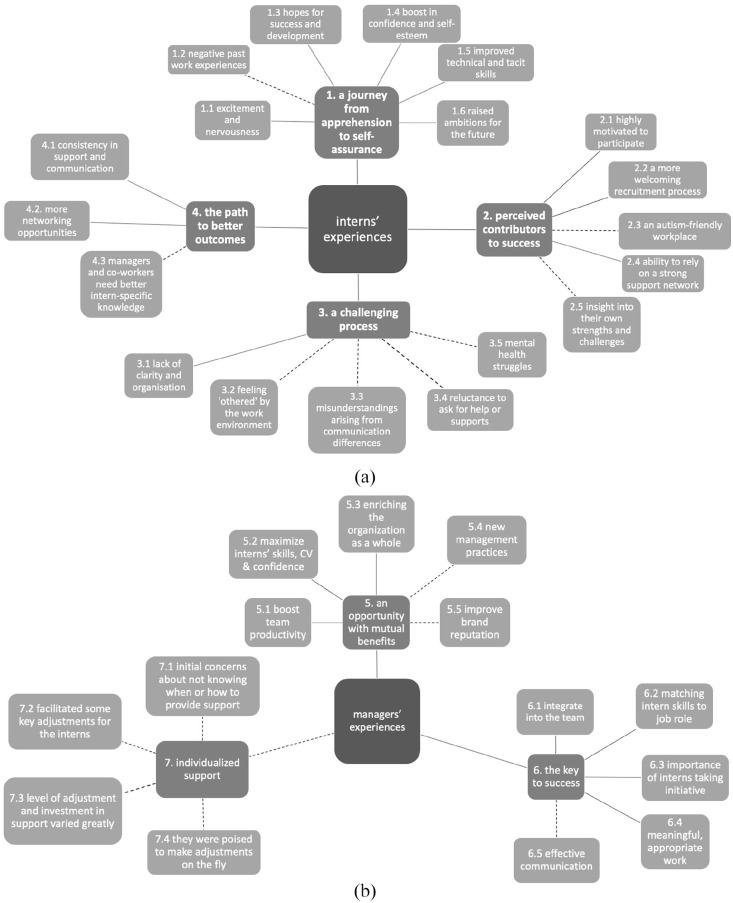
(a) Diagram of themes and subthemes relating to interns’ experiences (solid lines indicate themes shared by autistic and non-autistic participants. Dashed lines indicate themes raised only by autistic participants). (b) Diagram of themes and subthemes relating to managers’ experiences (solid lines indicate themes shared by managers of autistic and non-autistic interns. Dashed lines indicate themes raised only by managers of autistic interns).

Figure 1(a) shows the themes and subthemes for autistic and non-autistic interns’ journey through the internship. Interns reported overwhelmingly positive experiences of the process and reflected on factors that had led to their successes. While both groups of interns encountered challenges, a number of issues were identified specifically by the autistic interns. See Supplementary Materials for a full list of themes and subthemes, with example quotes.

##### A journey from apprehension to self-assurance

Autistic and non-autistic interns alike showed the development of increased confidence and improved skills across the internship process. There were a number of areas of apprehension in advance of beginning the programme, with both autistic and non-autistic interns expressing feelings of *excitement and nervousness (subtheme 1.1)*: ‘I’m actually really excited. I’m obviously a bit nervous as well, just it’s more the fear of the unknown’ (Non-Autistic Intern 14, henceforth NA-I14). The interns shared a sense of uncertainty about the work involved particularly regarding what they would be asked to do by their managers. They also revealed that they had not been given much information prior to the start of the internship, which contributed to this uncertainty. For example, one non-autistic intern shared, ‘I’m not actually quite sure what that involves. I’ve asked . . . my manager, but yeah I’m looking forward to finding out’ (NA-I15).

While both groups of interns spoke about feeling apprehensive, only the autistic interns referred to *negative past work experiences (subtheme 1.2)* that may have led to their apprehension about starting the internship: ‘Basically, I got about two or three assessment centres but I was rejected after the assessment centre’ (Autistic Intern 7, henceforth A-I7).

Nevertheless, autistic and non-autistic interns shared their *hopes for success and development through the internship (subtheme 1.3)*. One autistic intern stated,

It’s the fact that I’ll be doing something completely different to what I’ve been doing on a day to day basis. And hopefully I’ll find it, I’ll be given some challenging work to really kind of get my teeth into and some good problem solving. Because at the moment in my previous job I never really . . . I never really felt challenged, so I’d really like to be challenged. (A-I4)

These hopes materialised, with the autistic and non-autistic interns making frequent references to their achievement and personal development. Both groups mentioned *improved technical and tacit skills (subtheme 1.4)*:

I mean, for three months, I’ve managed to gain experience which is absolutely priceless and I gained, I’ve not only felt like I’ve further improved on skills I’ve gained before joining this internship, but I feel like I’ve gained lots of new and different skills I could’ve never thought I would have achieved. It’s been absolutely great. (A-I8)

Non-autistic interns spoke about developing a specific skill, saying, ‘We got some really excellent training on presentation skills in the internship, which was useful’ (NA-I1). Another success was the *boost in confidence and self-esteem* (subtheme 1.5) gained from the internship, which was common to both the autistic and non-autistic interns. Both groups made references to impressing their supervisors or colleagues and feeling more confident in the workplace once they had completed the internship.

Both the autistic and non-autistic interns ended the internship with *raised ambitions for the future (subtheme 1.6)*. Autistic interns suggested, ‘My goals and plan is certainly to stay in DB for as long as possible, because DB has opened many doors for me they can’t possibly imagine so’ (A-I8). Similarly, non-autistic interns spoke about how the internship would help them find jobs in the future: ‘it’s helped me so much because the experiences that I’ve kind of got from this internship and the responsibilities that I’ve had . . . if you say you’ve worked for an investment bank it’s quite impressive’ (NA-I11).

##### Perceived contributors to success

The interns from both the autism-specific and general internship schemes identified contributors to their successful journey through the internship and, potentially, the success of future internship programmes. These related both to personal characteristics of the interns themselves and aspects of the scheme and wider DB organisation.

Autistic and non-autistic interns spoke about being *highly motivated to participate in the scheme (subtheme 2.1)* due to the company reputation and prestige of the internship: ‘I know that Deutsche Bank are huge and they’re global . . . and the kind of stuff I’ll be working on will really impact people’ (NA-14).

For autistic and non-autistic interns alike, a *more welcoming recruitment process (subtheme 2.2)* compared to their past job-seeking experiences, gave them positive first impressions of DB and the internship schemes: ‘When I came in for both my interviews, both of them were really, really nice. Very accommodating’ (A-I11). Although the non-autistic interns had an additional step to their recruitment process (i.e. an assessment day) and more formal interviews, they also expressed positive feedback.

The autistic interns also talked specifically about being attracted to DB as *an autism-friendly workplace (subtheme 2.3)*: ‘Basically, I really like this company because . . . it was really friendly to autistic people and not many other companies are as friendly to autistics as Deutsche Bank are’ (A-I13). The autistic interns spoke about the positive impact of diagnostic disclosure within the programme. Joining an autism-specific internship scheme meant that their autism diagnosis was known to their managers and co-workers prior to them joining the company. They spoke about the positive impact of this diagnostic disclosure: ‘I was very excited in the sense that this is going to be specialised in something different, and obviously people are aware about my disability, which gives me a sense of a little bit of liberty’ (A-I12). This was borne out by examples of flexible work hours and adjustments to the physical work environment which benefitted the autistic interns in particular: ‘My manager gave me his seat by the window so that helped because I do like looking out the window as opposed to like seeing an office’ (A-I11).

When considering factors that related to the individual intern, rather than the organisation, both autistic and non-autistic interns spoke about *the ability to rely on a strong support network (subtheme 2.4)*. For the autistic interns, this predominantly included family members, while the non-autistic interns spoke about a wider network that included family, friends and other acquaintances:

I have a good relationship with my parents, so if something comes up, I’ll speak to them. I have a group of friends, I have other friends actually who are doing similar internships so, they may have experienced something similar as well so it may be good to speak to them. (NA-I9)

One theme unique to the autistic interns was their *insight into their own strengths and challenges (subtheme 2.5)*, both personal and professional. They spoke about an awareness of the skills they had (‘I’m organised. I’m a quick learner. I’m very honest, and I’m straight to the point’ (A-I6)) and especially about those they needed to develop: ‘My main weakness would be, I would say, my interpersonal skills in terms of how I am perceived by others . . . so, from my experience, these are my main issues’ (A-I2).

##### Challenges encountered along the way

Although the internship programme was overwhelmingly positive for both groups, the autistic and non-autistic interns spoke about a number of challenges they had encountered as they moved through the process. Both groups spoke about how *lack of clarity and organisation (subtheme 3.1)*, particularly in how managers communicated with the interns and day-to-day activities were scheduled, was a recurring challenge during the internship. One autistic intern stated, ‘I guess one thing which a number of the interns have talked about is . . . a lot of things have been not that well-organised. So, they’re things like last minute changes’ (A-I1).

Although both groups encountered difficult situations during the internship, the autistic interns experienced far more challenges that were unique to them. One challenge was the autistic interns *feeling ‘othered’ by the work environment (subtheme 3.2)*, or being made more aware of their differences in this particular setting. Autistic interns also spoke of *misunderstandings arising from communication differences (subtheme 3.3;* ‘it really put my head in a downward spiral, and then a couple of days I had a word with him about it and he said, ‘ah, that was my fault, I shouldn’t have worded it the way I did’ (A-I8)), and a certain *reluctance to ask for help or supports (subtheme 3.4)*, which stemmed from feeling worried about the negative perceptions of others: ‘Sometimes I do struggle with asking for help because I kind of feel a bit stupid having to ask sometimes’ (A-I4). In addition, *mental health struggles (subtheme 3.5)* posed a challenge specific to the autistic interns, with anxiety being a prominent mental health issue associated with aspects of work: ‘The anxiety or the stress really got to me. When I’m at a point where I’m not able to think, or my mind just goes blank or I shut down, and I’m not able to perform my daily tasks . . .’ (A-I3). For some, the anxiety was linked to the social demands of the role: ‘For me, the challenge was that I felt quite anxious when I was invited in a meeting’ (A-I7). For others, the anxiety was linked to the communication challenges outlined above: ‘it’s miscommunication like that that could cause me severe anxiety’ (A-I8). Encouragingly, some interns reported receiving support from colleagues and said that mentors had helped them better understand their feelings in such situations.

##### The path to better outcomes

Reflecting on their experiences through the internship, autistic and non-autistic interns shared their suggestions for how the process could be improved for future iterations of the scheme. Both groups of interns identified more *consistency in support and communication (subtheme 4.1)* as necessary for future success. For example, interns spoke about their need for a specific person within the company that they could rely on for help and clearer communication from the organisers of the scheme. *More networking opportunities for interns (subtheme 4.2)* were also flagged as one area in which the internship schemes could improve, with both autistic and non-autistic interns stating that they would have benefitted from opportunities to meet other people from different departments: ‘I really would have liked the opportunity to kind of spend a day shadowing a completely different division just to kind of broaden out that network’ (NA-I12).

For autistic interns, a key recommendation was that *managers and co-workers need better intern-specific knowledge (subtheme 4.3)* and felt that the general autism training was not enough to prepare their managers and co-workers for the scheme: ‘I think it would have been better for the intern to be involved or at least for the people delivering the training to know what they were training for’ (A-I3). Company training and management strategies specific to the intern would greatly improve the internship, as managers and co-workers would be given the opportunity to learn more about the autistic interns themselves.

#### Managers’ experiences through the internship programme

The interviews revealed many themes common to managing both autistic and non-autistic interns, and some which were specific to the managers of autistic interns (see Figure 1(b)).

##### An opportunity with mutual benefits

In advance of embarking on the scheme, all hiring managers hoped that the interns would find the internship a meaningful rewarding experience that could help *boost team productivity (subtheme 5.1); maximise interns’ skills, CV and confidence (subtheme 5.2)* and create a mutually beneficial environment *enriching the organisation as a whole (subtheme 5.3)*:

It’s just a good experience for people to learn, or to, yeah . . . learn and work with someone with autism, if they’re aware, and how they might need to adapt their personal communication styles to their approach in dealing with an employee, or even a manager, with autism. (A-HM7)

Managers of autistic interns, however, also anticipated additional benefits, specifically that it was an opportunity to learn *new management practices (subtheme 5.4)* and to *improve brand reputation (subtheme 5.5)* of the organisation as a whole. Specifically, there was recognition that the autism scheme was innovative, and as such it plays a role in breaking down barriers for future autistic applicants.

Encouragingly, these hopes mapped onto the internship outcomes. Managers reflected positively on how interns exceeded expectations, overcame difficulties encountered, got up to speed quickly and acquired new skills:

It’s gone pretty well for us. I’ve managed to secure an extension for [intern], so he’s going to be with us at least to the end of the year, so I think that indicates that it has been successful, and I’m willing to extend his stay with us. (A-HM12)

Managers of autistic interns also reported that working with the autistic intern benefitted the wider team. In particular, they reported that questioning their own taken-for-granted assumptions about communication and management practice to accommodate autistic styles of processing and interacting made them a better all-round manager. These effects were not limited to managers, but also benefitted co-workers as well:

I think the team have learnt those skill sets with [intern], you can’t just talk to him like he’s your best friend, you actually need to think about how you’re going to structure what you say, how are you going to structure what you want him to do and then acknowledge that he’s understood what you’ve said and that he will get on with what you want to do. So, I guess it helps our individual team with self-development in their personal skills. (A-HM2)

The positive difference observed throughout the internship was reflected in how some team members, who were initially anxious about how to support an autistic intern, reported that such concerns were quickly ameliorated.

##### The key to success

Managers of both groups identified a small number of key factors that they felt were crucial to a successful internship. First, managers of autistic and non-autistic interns reflected on how well their intern would *integrate into the team (subtheme 6.1)* and wider organisational culture. This was a topic for concern in advance of the internship starting (‘I guess I would be disappointed if they just sort of sat at their computer and (not) acknowledge everyone else, and didn’t socialise. We’re not a group of individuals, we’re a team’ – NA-HM3) and an area of success that was celebrated at its conclusion: ‘he integrated with the team, integrated with the department’ (A-HM2) Where integration was less successful, this was attributed to an issue in *matching intern skills to the job role (subtheme 6.2)*: ‘they weren’t a great fit, it’s basically because they didn’t have a bunch of technical skills that are mandatory for our group and the quality of the work reflected that’ (NA-HM3) Managers of both groups recommended that care be taken in the future when placing interns:

Well, I’d certainly review the roles available and ensure that they’re placed in the correct place because I could imagine some days [intern] must have been a bit frustrated because he’s not doing as much as he could do, especially coming from a mathematical background and stuff, it didn’t feel to me as though we were challenging him enough. (A-HM5)

Second, managers highlighted the *importance of interns taking initiative (subtheme 6.3)*. In advance of the internship beginning, managers of autistic and non-autistic interns stressed that this was vital if the intern was to be successful in the team. In keeping with this, both groups of managers felt that less successful outcomes were seen when interns did not take initiative:

I would have liked to have seen a little bit more effort to get involved [. . .] being a little bit more proactive and realising that you’ve got to put yourself out there a bit. Not everyone’s going to lay it out in front of you all the time, you’ve actually got to make a bit of an effort. (NA-HM2)

Third, managers recognised their own role in ensuring that their intern had *meaningful, appropriate work (subtheme 6.4)* to do: ‘I think the only thing I am concerned about is making sure that I have enough for him to do, because it is quite difficult to package things up isn’t it, for a short period of time?’ (A-HM11). At the conclusion of the scheme, managers of autistic interns reflected that in future, they would aim to have structured tasks planned in advance:

I’d be a bit more organised when he started, and have my initial tasks, and initial couple of weeks planned out for him better, so that I could give him some specific work that would get him to speed, get him started, and up to speed, and settled in. (A-HM12)

For managers of autistic interns specifically, the need for *effective communication (subtheme 6.5)* was raised. For example, managers anticipated challenges when ensuring that instructions were correctly understood:

in terms of the communication, yeah, that was the one that I really didn’t know what to expect so I didn’t know how well I’d be able to communicate and I’m aware that I need to set some quite specific goals, you know, in giving out tasks. (A-HM12)

Managers were also anxious about whether autistic interns would speak up about their concerns and let them know when more support was required and anticipated more time and effort would be required to communicate successfully.

During the internship, these concerns materialised and managers spoke about adapting their own communication style to address the issues they encountered:

It was hard sort of to communicate verbally and exactly find out whether I was being understood or not, or whether what I was . . . It was hard to say. You know, I was giving him a [a task] and I couldn’t tell whether I was making total sense when I was like explaining something that should be as obvious as that wall over there, or whether he wasn’t getting one word I was saying at all. So I had to definitely adapt that style to say [. . .] ‘Does that sound easy, medium or hard?’ He always said medium. I didn’t know exactly, yeah, whether he’d got it or not. (A-HM1)

##### Individualised support

Managers of autistic interns raised concerns about providing the right level of support, using the right management style, identifying and implementing appropriate adjustments, and ensuring equitable treatment of all employees: ‘in reality I probably wouldn’t want to treat my intern different from any other intern I have worked with’ (A-HM6). In some cases, *concerns about not knowing when or how to provide support (subtheme 7.1)* were linked to a lack of confidence around their autism knowledge. Managers expressed uncertainty about what to expect, being cautious about having too many expectations and also uncertainty about understanding autism and how to implement training that was provided:

I think, you know, my knowledge of autism and my knowledge of this individual’s capabilities are relatively limited to, you know, an interview, so I think to be too ambitious and kind of create a very detailed plan for the full 12 weeks. (A-HM4)

Although managers often reported using their knowledge about autism provided via training before the start of the internship, a number of managers also reflected that there were further opportunities to enhance co-worker knowledge of autism around the autistic intern: ‘People who are unaware of autism and [intern]’s issue they were probably a bit like I don’t understand, he keeps disappearing and locking himself in a meeting room’ (A-HM2).

Despite these worries, the managers of autistic interns had *facilitated some key adjustments for their interns (subtheme 7.2)*. Many of these related to the physical work environment (such as reserving desks within a hot-desking environment for autistic interns, making use of equipment such as noise-cancelling headphones to minimise distractions) or offering greater flexibility in working pattern, for example shifting working hours to avoid rush hour traffic. In addition, managers reported needing to offer additional support with time management:

managing his time effectively to make sure that a) he’s not sitting there, not doing anything and bored, or b) not distracted and going off down a path of, I’ve said, “Oh, go and look into that”, just as a background task, that he might dedicate all of his time to that. (A-HM12)

In some cases, a lack of resources, or the nature of the work environment, made it challenging to implement the necessary support:

Part of the nature of the team we have in London in my particular team and the work that’s here, I think it was challenging for us sometimes to probably spend as much time with [intern] and give enough sort of repeatable tasks that would be then easy for him, easier for him to pick up. (A-HM10)

Managers recommended ensuring that there were appropriate resources in place when beginning a placement with an autistic intern: ‘make sure that you’ve got enough resources in place and the time to constantly be working closely’ (A-HM11).

That said, the *level of adjustments required, and the investment required to support the intern, could vary greatly (subtheme 7.3)*, with some interns not needing much support at all. Managers recognised this variable need: ‘I think the main adjustment is to recognise the individual, what their needs are, and to be proactive at observing, and obviously tailoring, and flexing, and being agile, so we’re sure that we’re able to provide that’ (A-HM7).

The managers also demonstrated that *they were poised to make adjustments on the fly (subtheme 7.4)*, which they reported to be a positive management strategy:

He was sitting next to someone one day and he asked me if I could hear this buzzing sound (laughs) and it turned out it was from . . . the guy next to us charged his mobile phone in this particular socket, not any other one but this particular one, it sort of made this high frequency buzz which he could hear and so on. (Laughing) Yeah, and we sort of asked him to just put it into the next socket and that was fine. (A-HM1)

## Discussion

Internships within a high-pressured corporate environment could potentially pose a number of challenges for autistic and non-autistic interns alike. The central aim of this study was to understand how workplace experiences of autistic interns differed from their non-autistic peers, and how this changed through the course of the internship. The results highlighted many commonalities between autistic and non-autistic interns’ journeys through the programme, especially around motivation to participate in the scheme, the development of skills and self-confidence and, from a manager’s viewpoint, aspiration that the interns will play a role in enhancing productivity and the wider company culture. While previous studies have shown that aspirations around the role of employment in elevating skills, independence and making a meaningful contribution to the workplace, are important values for autistic people (Sosnowy et al., 2018), the comparative approach of this study highlights that such aspirations are not necessarily specific to autistic people. While much of the prior research on autistic employment considers only autistic individuals, our approach allowed a direct comparison to be made between autistic and non-autistic colleagues and challenge, at least in part, the assumption that autistic experiences diverge from non-autistic experiences in the workplace.

That said, there were some areas in which autistic interns’ experiences were unique. In one such area, autistic interns were more likely to report prior negative workplace experiences, consistent with research on autistic adults’ experiences of frequent job changes and periods of unemployment (Ohl et al., 2017). Past experiences can play an important role in contributing to one’s own awareness of strengths, weaknesses and expectations about new job roles, and this was reflected in the significantly lower WS-Ei scores regarding confidence for autistic interns compared with non-autistic interns at the start of the internship. Qualitative analysis also showed that managers of autistic interns did not discuss the potential for negative past employment experiences to impact current performance, despite it being more prominent in reports from autistic interns themselves. Differing levels of awareness about past challenges and its impact on confidence between autistic interns and their managers suggest that there may still be opportunities for improving proactive support to help autistic interns overcome potential challenges with confidence.

A second area that was perceived to be more challenging for autistic interns was mental health and well-being. Mental health conditions, such as anxiety and depression, have been shown to be much higher in autistic populations (Cassidy et al., 2018; Lai et al., 2019) compared with those who are not autistic (Au-Yeung et al., 2019). In the present study, anxiety was a common feature reported by *all* interns in advance of starting the internship. At the conclusion of the internship, however, only the autistic interns highlighted the impact of anxiety throughout the duration of the scheme. Further proactive awareness about the wider health and well-being contexts of autistic interns should be a key consideration for managers during future programmes, particularly given the communication challenges autistic people experience in speaking up about how they may be feeling (Cage et al., 2018; Heasman, 2017).

A third area focused on the communication challenges experienced specifically by autistic interns – consistent with previous reports on autistic barriers in the workplace (Black et al., 2020; Howlin, 2000). Through comparing interns with their managers, however, results indicated that these communication challenges were often two-sided. For autistic interns, misunderstandings arose from different communication styles, such as a preference for written instructions via email over verbal instructions. Such strategies help to ameliorate autistic difficulties in interpreting implicit social cues (Schuwerk et al., 2015). But it was also observed that there were opportunities for the organisation to clarify further their existing processes of onboarding an intern into a new role and team. For example, some meetings involved rapid discussions that could be challenging for autistic interns who required longer processing time. Nevertheless, there were efforts made to account for different communication styles that can cause misunderstandings for autistic people (Milton, 2012), such as adapting how instructions were communicated so that there were no competing priorities. Greater flexibility in communication style has been identified as a central feature of successful collaboration between autistic people (Heasman & Gillespie, 2019b) and the adaptations made by managers reflect how non-autistic people can potentially learn from autistic social feedback (Heasman & Gillespie, 2019a).

When considering workplace performance, the frequency of successful task comprehension and planning and level of independence when dealing with distractions were scored significantly lower by managers of autistic interns compared with non-autistic interns. This may be in part explained by the higher average self-reported ADHD traits of autistic interns. However, managers were uniformly positive about the frequency and independence with which tasks were successfully performed: the ratings did not significantly differ between managers of autistic and non-autistic interns. This dynamic is also reflected in the qualitative reports from managers post-internship. Although there were some challenges associated with communicating with autistic interns, and knowledge about autism among team members, there were also many positive cases where the autistic interns had successfully integrated into the team, exceeding expectations in the process.

The comparative and longitudinal approaches adopted in this study have shown how autistic talent can also flourish in ways that are similarly observed by managers of non-autistic interns. Autistic interns showed evidence of accurately self-evaluating areas for improvement around task comprehension and planning, which compared with the rating of WPQ scores provided by managers. The potential for autistic interns to accurately self-evaluate areas for improvement contrasts with the view that autistic people are limited in their ability to reflect on themselves from the social positions of others (Frith & De Vignemont, 2005). These findings therefore add to our understanding of the strengths and the potential for positive impact that autistic people bring to the workplace (Anderson et al., 2021; Holwerda et al., 2013).

Regardless of a diagnosis, managers reflected that recruitment processes needed to match candidate skills to job roles effectively. This process is also shaped, however, by the organisational culture. In particular, managers of autistic interns reported a concern about treating autistic interns differently from other interns, a sentiment observed in prior research (Remington & Pellicano, 2019). This highlights a tension between a focused effort to tailor aspects of recruitment to be more accessible for hard-to-access populations and, at the same time, a desire to treat all employees equitably. Understanding how to navigate this dynamic, such as bringing together managers and autistic employees to discuss issues, would be an important step towards establishing a successful environment for supporting autistic talent within an organisation. The need for a tailored approach was echoed by many of the autistic interns who highlighted a lack of knowledge around their specific needs and emphasised the value of having interns and managers work together to achieve better outcomes. This is in keeping with research demonstrating the importance of customised employment where job descriptions are modified in line with the employee’s profile of strengths and challenges (Wehman et al., 2016). The managers of autistic interns in our study, however, reported willingness to make necessary adjustments but reflected on difficulties knowing when or how to provide support, in some cases due to the great variety in level of support needed across individuals.

### Limitations

This study is not without its limitations. First, participants were taking part within a well-established scheme, thus autistic interns in other organisations may face greater challenges associated with lower organisational awareness of autism. Second, the autistic interns who took part were slightly older than the non-autistic interns. This may have contributed to some of the experiences that were shared uniquely by this group, for example, the increased level of negative past work experiences. Likewise, the autistic interns were predominantly male, compared to the equal split between males and females in the non-autistic group. While this sampling bias likely reflects the higher autism diagnosis rates seen for males compared to females, future research should aim, where possible, to consider the experiences of more closely matched autistic and non-autistic employees. Third, the small sample size limits the conclusions that can be drawn from the quantitative data, especially with regard to interpreting the absence of group differences (e.g. with respect to manager reports of intern performance). Although these conclusions mapped onto our qualitative results, research with larger participant groups is necessary to confirm the quantitative findings of the present study. Finally, the participation rates for the autism-specific and general DB internship programme were very different. All interns and their managers from the autism-specific scheme took part in the research, meaning that the entire range of experiences was fully represented in the present study. With respect to the general scheme, a self-selecting group volunteered to take part in the research: 15 interns (equivalent in group size to the autistic participants) but only five managers (from 180). It was perhaps inevitable that engagement with the research from those on the autism-specific scheme would be greater, but this potential recruitment bias somewhat limits the generalisability of the findings with respect to managers of the non-autistic interns.

## Conclusion

Our findings highlight unique workplace benefits and challenges specific to autistic interns compared with non-autistic interns. Autistic interns experienced a greater range of negative past employment experiences, more residual anxiety throughout the internship and greater difficulties in communicating with managers than their non-autistic peers. Nevertheless, autistic interns also experienced notable successes comparable to non-autistic interns, including increased self-confidence and contributing to the productivity of the team. Moreover, autistic people also brought added advantages in terms of enhancing the communication and management practices of managers as a whole. Likewise, although managers of autistic interns reported a greater range of concerns associated with integrating their intern into the team culture, there were many cases of this process being navigated successfully. Further research that examines the effectiveness of specific workplace adjustments over extended periods of time can enable managers and organisations to provide more targeted support to maximise the development of employee well-being and talent.

## Supplemental Material

sj-docx-1-aut-10.1177_1049732320931430 – Supplemental material for Experiences of autistic and non-autistic individuals participating in a corporate internship scheme Click here for additional data file. Supplemental material, sj-docx-1-aut-10.1177_1049732320931430 for Experiences of autistic and non-autistic individuals participating in a corporate internship scheme by Anna Remington, Brett Heasman, Anna Melissa Romualdez and Elizabeth Pellicano in Autism

sj-docx-2-aut-10.1177_1049732320931430 – Supplemental material for Experiences of autistic and non-autistic individuals participating in a corporate internship scheme Click here for additional data file. Supplemental material, sj-docx-2-aut-10.1177_1049732320931430 for Experiences of autistic and non-autistic individuals participating in a corporate internship scheme by Anna Remington, Brett Heasman, Anna Melissa Romualdez and Elizabeth Pellicano in Autism

sj-docx-3-aut-10.1177_1049732320931430 – Supplemental material for Experiences of autistic and non-autistic individuals participating in a corporate internship scheme Click here for additional data file. Supplemental material, sj-docx-3-aut-10.1177_1049732320931430 for Experiences of autistic and non-autistic individuals participating in a corporate internship scheme by Anna Remington, Brett Heasman, Anna Melissa Romualdez and Elizabeth Pellicano in Autism

## Notes

1. In the United Kingdom, university degree results are classified as First-Class Honours (First or 1st, 70% and above), Upper Second-Class Honours (2:1, 2.i, 60%–70%), Lower Second-Class Honours (2:2, 2.ii, 50%–60%) or Third-Class Honours (Third or 3rd, 40%–50%).

## References

[bibr1-13623613211025115] Anderson C. Butt C. Sarsony C. (2021). Young adults on the autism spectrum and early employment-related experiences: Aspirations and obstacles. Journal of Autism and Developmental Disorders, 51, 88–105. 10.1007/s10803-020-04513-4 32356082

[bibr2-13623613211025115] Au-Yeung S. K. Bradley L. Robertson A. E. Shaw R. Baron-Cohen S. Cassidy S. (2019). Experience of mental health diagnosis and perceived misdiagnosis in autistic, possibly autistic and non-autistic adults. Autism, 23(6), 1508–1518. 10.1177/1362361318818167 30547677

[bibr3-13623613211025115] Baldwin S. Costley D. Warren A. (2014). Employment activities and experiences of adults with high-functioning autism and Asperger’s disorder. Journal of Autism and Developmental Disorders, 44(10), 2440–2449. 10.1007/s10803-014-2112-z 24715257

[bibr4-13623613211025115] Barnhill G. P. (2007). Outcomes in adults with Asperger syndrome. Focus on Autism & Other Developmental Disabilities, 22(2), 116–126. 10.1177/10883576070220020301

[bibr5-13623613211025115] Billstedt E. Gillberg C. Gillberg C. (2005). Autism after adolescence: Population-based 13- to 22-year follow-up study of 120 individuals with autism diagnosed in childhood. Journal of Autism and Developmental Disorders, 35(3), 351–360. 10.1007/s10803-005-3302-5 16119476

[bibr6-13623613211025115] Black M. H. Mahdi S. Milbourn B. Scott M. Gerber A. Esposito C. . . . Girdler S. (2020). Multi-informant international perspectives on the facilitators and barriers to employment for autistic adults. Autism Research, 13(7), 1195–1214. 10.1002/aur.2288 32170919

[bibr7-13623613211025115] Braun V. Clarke V. (2019). Reflecting on reflexive thematic analysis. Qualitative Research in Sport, Exercise and Health, 11(4), 589–597. 10.1080/2159676X.2019.1628806

[bibr8-13623613211025115] Buescher A. V. S. Cidav Z. Knapp M. Mandell D. S. (2014). Costs of autism spectrum disorders in the United Kingdom and the United States. JAMA Pediatrics, 168(8), 721–728. 10.1001/jamapediatrics.2014.210 24911948

[bibr9-13623613211025115] Cage E. Di Monaco J. Newell V. (2018). Experiences of autism acceptance and mental health in autistic adults. Journal of Autism and Developmental Disorders, 48(2), 473–484. 10.1007/s10803-017-3342-7 29071566 PMC5807490

[bibr10-13623613211025115] Cassidy S. Bradley L. Shaw R. Baron-Cohen S. (2018). Risk markers for suicidality in autistic adults. Molecular Autism, 9, 42. 10.1186/s13229-018-0226-4 30083306 PMC6069847

[bibr11-13623613211025115] Chen J. L. Leader G. Sung C. Leahy M. (2015). Trends in employment for individuals with autism spectrum disorder: A review of the research literature. Review Journal of Autism and Developmental Disorders, 2(2), 115–127. 10.1007/s40489-014-0041-6

[bibr12-13623613211025115] Connolly P. Bacon N. Wass V. Hoque K. Jones M. (2016). ‘Ahead of the arc’—A contribution to halving the disability employment gap. The All Party Parliamentary Group on Disability. https://www.disabilityrightsuk.org/sites/default/files/pdf/AheadoftheArc110518.pdf

[bibr13-13623613211025115] Constantino J. N. Gruber C. P. (2012). Social Responsiveness Scale–Second Edition (SRS-2). Western Psychological Services.

[bibr14-13623613211025115] Department of Health. (2014). Fulfilling and rewarding lives: The strategy for adults with autism in England. https://webarchive.nationalarchives.gov.uk/20130104203954/http://www.dh.gov.uk/en/Publicationsandstatistics/Publications/PublicationsPolicyAndGuidance/DH_113369

[bibr15-13623613211025115] Frenette A. (2013). Making the intern economy: Role and career challenges of the music industry intern. Work and Occupations, 40(4), 364–397. 10.1177/0730888413504098

[bibr16-13623613211025115] Frith U. De Vignemont F. (2005). Egocentrism, allocentrism, and Asperger syndrome. Consciousness and Cognition, 14(4), 719–738. 10.1016/j.concog.2005.04.006 15996486

[bibr17-13623613211025115] Gal E. Landes E. Katz N. (2015). Work performance skills in adults with and without high functioning autism spectrum disorders (HFASD). Research in Autism Spectrum Disorders, 10, 71–77. 10.1016/j.rasd.2014.10.011 25735411

[bibr18-13623613211025115] Griffith G. M. Totsika V. Nash S. Hastings R. P. (2012). ‘I just don’t fit anywhere’: Support experiences and future support needs of individuals with Asperger syndrome in middle adulthood. Autism, 16(5), 532–546. 10.1177/1362361311405223 21610188

[bibr19-13623613211025115] Heasman B. (2017, July 31). Employers may discriminate against autism without realising. LSE Business Review. http://eprints.lse.ac.uk/id/eprint/83831

[bibr20-13623613211025115] Heasman B. Gillespie A. (2019a). Learning how to read autistic behavior from interactions between autistic people. Behavioral and Brain Sciences, 42, Article e93. 10.1017/S0140525X18002364

[bibr21-13623613211025115] Heasman B. Gillespie A. (2019b). Neurodivergent intersubjectivity: Distinctive features of how autistic people create shared understanding. Autism, 23(4), 910–921. 10.1177/1362361318785172 30073872 PMC6512057

[bibr22-13623613211025115] Hedley D. Uljarevi M. Cameron L. Halder S. Richdale A. Dissanayake C. (2017). Employment programmes and interventions targeting adults with autism spectrum disorder: A systematic review of the literature. Autism, 21(8), 929–941. 10.1177/1362361316661855 27542395

[bibr23-13623613211025115] Hendricks D. (2010). Employment and adults with autism spectrum disorders: Challenges and strategies for success. Journal of Vocational Rehabilitation, 32(2), 125–134. 10.3233/JVR-2010-0502

[bibr24-13623613211025115] Hillier A. Campbell H. Mastriani K. Izzo M. V. Kool-Tucker A. K. Cherry L. Beversdorf D. Q. (2007). Two-year evaluation of a vocational support program for adults on the autism spectrum. Career Development for Exceptional Individuals, 30(1), 35–47. 10.1177/08857288070300010501

[bibr25-13623613211025115] Holland J. (2007). Qualitative longitudinal research: Exploring ways of researching lives through time. Real Life Methods Node of the ESRC National Centre for Research Methods Workshop held at London South Bank University. https://citeseerx.ist.psu.edu/viewdoc/download?doi=10.1.1.485.7802&rep=rep1&type=pdf

[bibr26-13623613211025115] Holwerda A. van der Klink J. J. L. de Boer M. R. Groothoff J. W. Brouwer S. (2013). Predictors of sustainable work participation of young adults with developmental disorders. Research in Developmental Disabilities, 34(9), 2753–2763. 10.1016/j.ridd.2013.05.032 23792372

[bibr27-13623613211025115] Howlin P. (2000). Outcome in adult life for more able individuals with autism or Asperger syndrome. Autism, 4(1), 63–83. 10.1177/1362361300004001005

[bibr28-13623613211025115] Katz N. Dejak I. Gal E. (2015). Work performance evaluation and QoL of adults with High Functioning Autism Spectrum Disorders (HFASD). Work, 51(4), 887–892. 25735411 10.3233/WOR-152001

[bibr29-13623613211025115] Kessler R. C. Adler L. Ames M. Demler O. Faraone S. Hiripi E. . . . Walters E. E. (2005). The World Health Organization Adult ADHD Self-Report Scale (ASRS): A short screening scale for use in the general population. Psychological Medicine, 35(2), 245–256. 10.1017/s0033291704002892 15841682

[bibr30-13623613211025115] Kroenke K. Spitzer R. L. WIlliams J. B. (2001). The PHQ-9: Validity of a brief depression severity measure. Journal of General Internal Medicine, 16(9), 606–613. 10.1046/j.1525-1497.2001.016009606.x 11556941 PMC1495268

[bibr31-13623613211025115] Lai M.-C. Kassee C. Besney R. Bonato S. Hull L. Mandy W. . . . Ameis S. H. (2019). Prevalence of co-occurring mental health diagnoses in the autism population: A systematic review and meta-analysis. The Lancet. Psychiatry, 6(10), 819–829. 10.1016/s2215-0366(19)30289-5 31447415

[bibr32-13623613211025115] Lorenz T. Frischling C. Cuadros R. Heinitz K. (2016). Autism and overcoming job barriers: Comparing job-related barriers and possible solutions in and outside of autism-specific employment. PLOS ONE, 11(1), Article e0147040. 10.1371/journal.pone.0147040 PMC4713226 26766183

[bibr33-13623613211025115] Lorenz T. Heinitz K. (2014). Aspergers – Different, not less: Occupational strengths and job interests of individuals with Asperger’s syndrome. PLOS ONE, 9(6), Article e100358. 10.1371/journal.pone.0100358 PMC4065100 24950060

[bibr34-13623613211025115] Maenner M. J. Smith L. E. Hong J. Makuch R. Greenberg J. S. Mailick M. R. (2013). Evaluation of an activities of daily living scale for adolescents and adults with developmental disabilities. Disability and Health Journal, 6(1), 8–17. 10.1016/j.dhjo.2012.08.005 23260606 PMC3531884

[bibr35-13623613211025115] Mclaughlin M. E. Bell M. P. Stringer D. Y. (2004). Stigma and acceptance of persons with disabilities: Understudied aspects of workforce diversity. Group and Organization Management, 29(3), 302–333. 10.1177/1059601103257410

[bibr36-13623613211025115] Milton D. (2012). On the ontological status of autism: The ‘double empathy problem’. Disability & Society, 27(6), 883–887. 10.1080/09687599.2012.710008

[bibr37-13623613211025115] National Autistic Society. (2016). The autism employment gap – Too much information in the workplace. https://www.autism.org.uk/what-we-do/news/government-must-tackle-the-autism-employment-gap

[bibr38-13623613211025115] Noort M. C. Reader T. W. Gillespie A. (2019). Speaking up to prevent harm: A systematic review of the safety voice literature. Safety Science, 117, 375–387. 10.1016/j.ssci.2019.04.039

[bibr39-13623613211025115] Ohl A. Sheff M. G. Little S. Nguyen J. Paskor K. Zanjirian A. (2017). Predictors of employment status among adults with autism spectrum disorder. Work, 56(2), 345–355. 10.3233/WOR-172492 28211841

[bibr40-13623613211025115] Raelin J. A. (2010). The Work Self-Efficacy Inventory. Mind Garden. http://www.mindgarden.com/products/wsei.htm

[bibr41-13623613211025115] Remington A. Pellicano E. (2019). ‘Sometimes you just need someone to take a chance on you’: An internship programme for autistic graduates at Deutsche Bank, UK. Journal of Management & Organization, 25(4), 516–534. 10.1017/jmo.2018.66

[bibr42-13623613211025115] Richards J. (2012). Examining the exclusion of employees with Asperger syndrome from the workplace. Personnel Review, 41(5), 630–646. 10.1108/00483481211249148

[bibr43-13623613211025115] Rosenthal R. Rubin D. B. (2003). r equivalent: A simple effect size indicator. Psychological Methods, 8(4), 492–496. 10.1037/1082-989X.8.4.492 14664684

[bibr44-13623613211025115] Schuwerk T. Vuori M. Sodian B. (2015). Implicit and explicit theory of mind reasoning in autism spectrum disorders: The impact of experience. Autism, 19(4), 459–468. 10.1177/1362361314526004 24627427

[bibr45-13623613211025115] Scott M. Milbourn B. Falkmer M. Black M. Bölte S. Halladay A. . . . Girdler S. (2018). Factors impacting employment for people with autism spectrum disorder: A scoping review. Autism, 23(4), 869–901. 10.1177/1362361318787789 30073870

[bibr46-13623613211025115] Shattuck P. Narendorf S. C. Cooper B. Sterzing P. R. Wagner M. Taylor J. L. (2012). Postsecondary education and employment among youth with an autism spectrum disorder. Pediatrics, 129(6), 1042–1049. 10.1542/peds.2011-2864 22585766 PMC3362908

[bibr47-13623613211025115] Skevington S. Lotfy M. O’Connell K. (2004). The World Health Organization’s WHOQOL-BREF quality of life assessment: Psychometric properties and results of the international field trial. A report from the WHOQOL Group. Quality of Life Research, 13, 299–310. 10.1023/B:QURE.0000018486.91360.00 15085902

[bibr48-13623613211025115] Sosnowy C. Silverman C. Shattuck P. (2018). Parents’ and young adults’ perspectives on transition outcomes for young adults with autism. Autism, 22(1), 29–39. 10.1177/1362361317699585 29020791

[bibr49-13623613211025115] Spitzer R. L. Kroenke K. Williams J. B. W. Löwe B. (2006). A brief measure for assessing generalized anxiety disorder: The GAD-7. Archives of Internal Medicine, 166(10), 1092–1097. 16717171 10.1001/archinte.166.10.1092

[bibr50-13623613211025115] Suppiah V. Sandhu M. S. (2011). Organisational culture’s influence on tacit knowledge-sharing behaviour. Journal of Knowledge Management, 15(3), 462–477. 10.1108/13673271111137439

[bibr51-13623613211025115] Thomson R. Holland J. (2003). Hindsight, foresight and insight: The challenges of longitudinal qualitative research. International Journal of Social Research Methodology, 6(3), 233–244. 10.1080/1364557032000091833

[bibr52-13623613211025115] Wehman P. Brooke V. Brooke A. M. Ham W. Schall C. McDonough J. . . . Avellone L. (2016). Employment for adults with autism spectrum disorders: A retrospective review of a customized employment approach. Research in Developmental Disabilities, 53–54, 61–72. 10.1016/j.ridd.2016.01.015 26855048

[bibr53-13623613211025115] Wehman P. Schall C. M. McDonough J. Kregel J. Brooke V. Molinelli A. . . . Thiss W. (2014). Competitive employment for youth with autism spectrum disorders: Early results from a randomized clinical trial. Journal of Autism and Developmental Disorders, 44(3), 487–500. 10.1007/s10803-013-1892-x 23893098

[bibr54-13623613211025115] Willig C. (2013). Introducing qualitative research in psychology (3rd ed.). Open University Press.

[bibr55-13623613211025115] World Health Organization. (1996). WHOQOL-BREF: Introduction, administration, scoring and generic version of the assessment: Field trial version, December 1996. https://apps.who.int/iris/handle/10665/63529

