# Supplementary material for: Experiences of autistic and non-autistic individuals participating in a corporate internship scheme
Source: Autism. 2021 Jun 19;26(1):201–16. doi: 10.1177/13623613211025115 (PMC8750129; doi:10.1177/13623613211025115)
Supplement: sj-docx-1-aut-10.1177_1049732320931430 – Supplemental material for Experiences of autistic and non-autistic individuals participating in a corporate internship scheme [file sj-docx-1-aut-10.1177_1049732320931430.docx]

**Supplementary Materials A. Interview Schedules**

1. **Pre-internship Intern Interview Schedule**

| **Primary question** | **Probe questions** |
| --- | --- |
| Tell me a bit about yourself? | 1. Tell me a bit about what school was like – the good bits and the not-so-good bits. What sort of school did you go to? Did you get enough help if you needed it? Who helped you? |
|  | 1. Do you remember receiving your diagnosis of autism? How did you feel when you found out that you have autism? Is it something you talk about openly with others? |
|  | 1. Tell me about your time at university. What did you study? Did you complete your degree during 3 years or did you need to stop for any reason? Did you get any help/support during your time at university? |
|  | 1. Who helped you? Did you get enough help/support? |
|  | 1. When did you complete your university degree? What have you been doing in the meantime? |
| Tell me why you were interested in applying for the Deutsche Bank Work Placement internship? | 1. For those who haven’t had a period of employment |
|  | 1. How long have you been looking for a job? |
|  | 1. How many applications have you submitted? |
|  | 1. Have you had any interviews? |
|  | 1. Have you received any feedback from unsuccessful applications? |
|  | 1. Do you disclose that you are autistic on the applications? Why/why not? |
|  | 1. For those who have had jobs in the past |
|  | 1. Tell me about the jobs that you have had in the past. What worked well in those jobs? What problems did you experience? |
|  | 1. What are you hoping to do in the future? |
|  | 1. How did you hear about the Deutsche bank internship? |
|  | 1. How did you find the application process? |
|  | 1. How do you feel this internship will help you to achieve your goals? |
|  |  |
| Tell me a bit about what the internship will involve? | 1. How do you feel about it? |
|  | 1. Which aspects will you find exciting? |
|  | 1. Which things do you think you will find more challenging? |
|  |  |
|  | 1. What do you think you need to do well in the internship? |
|  | 1. Where do you think your strengths and weaknesses lie? |
|  |  |
| Let’s focus a bit on the possible challenges you might face during the internship? | 1. How much support do you think you will need? |
|  | 1. What is the one thing that you are most worried about? |
|  | 1. Have you let anyone at Deutsche Bank know about this? |
|  | 1. Who did you speak to and what did they say? |
|  | 1. Has anything been put in place to help you with this? |
|  | 1. The internship involves working with a team of other people – how do you generally get along with other people? |
|  | 1. How do you feel about asking people for help? |
|  | 1. What can you do to overcome any difficulties with asking for help? |
|  | 1. Do you have people you talk to when things get tough? |
|  | 1. What about family, do you get on well with them? Have they been supportive? |
|  | 1. Who do you live with at the moment? |
|  | 1. How are you going to find travelling to Deutsche Bank? |
|  |  |

1. **Pre-internship Manager Interview Schedule**

| **Primary question** | **Probe questions** |
| --- | --- |
| Tell me a bit about yourself | 1. Which area do you specialize in? |
|  |  |
|  | 1. How long have you worked with Deutsche Bank? |
|  | 1. Before the development of this internship programme, what did you know about autism? |
|  | 1. What are some of your previous experiences with individuals on the autistic spectrum? |
|  | 1. Have you worked with anyone autistic at Deutsche Bank? |
| Tell me a bit about the motivation behind and development of the internship programme for [autistic] graduates. | 1. Were you personally involved in the creation of the programme? |
|  | 1. How were line managers chosen for the programme? |
|  | 1. What is the main aim of the programme? |
|  | 1. What benefits/opportunities do you see for Deutsche Bank as an organisation? |
|  | 1. What benefits/opportunities do you see for the people working at Deutsche Bank? |
|  | 1. What are your hopes for the autistic interns themselves? What do you hope that they will get out of the programme? |
|  | 1. What would be your main indicator of ‘success’ for this programme? |
|  | 1. How many applications did you receive for the internship programme? Was it difficult to choose amongst them? |
|  |  |
|  |  |
| Tell me a bit about some of your expectations for the programme and the interns. | 1. How do you feel they will get on in terms of: |
|  | a. the quality of their work |
|  | 1. the social side of work |
|  | 1. practical things (e.g., getting to work, dress code etc.) |
| What about your apprehensions about the programme, if any? | 1. What do you think are some of things that will be the most challenging for you and your non-autistic colleagues with respect to the interns/internships? What are you most worried about? |
|  | 1. What do you think are some of things that will be the most challenging for the autistic interns? |
|  | 1. How much support do you think they will need? In what areas do you think they will need support? |
|  | 1. What accommodations, either personally or in the workplace, do you anticipate having to make to accommodate the autistic interns? |
|  |  |

1. **Post-internship Intern Interview Schedule**

|  |  |
| --- | --- |
| **Primary question** | **Probe questions** |
| Tell me a bit about how the internship went? | 1. How do you feel about it? |
|  | 1. What sort of tasks were you involved with? |
|  | 1. Which aspects did you enjoy? |
|  | 1. What did you do well in the internship? |
|  | 1. Where do you think your strengths and weaknesses lie? |
| Let’s focus a bit on any challenges you faced during the internship | 1. Which things did you find more challenging? (e.g., long hours, the work itself, the pressure, interacting with other people, planning the day) |
|  | 1. Did you tell anybody what you found challenging? What support were you given to help with this? |
|  | 1. How did you access this support? Did you ask for it? |
|  | 1. Was it enough? |
|  | 1. What else would have been helpful? |
|  | 1. Were any other adjustments made to help you through the internship? If so, what were they? If not, could something more have been done to help support you? |
|  | 1. Did you have people to talk to when things got tough? What about family, do you get on well with them? Have they been supportive? |
|  | 1. The internship involved working with a team of other people – how did you generally get along with them? Did you do anything socially with them during or outside of work time? How did you feel about asking people for help? |
|  | 1. How did you find travelling to Deutsche Bank (and living in London)? |
| Let’s talk a bit about the outcomes of the internship for you | 1. Now that the internship has finished, what are your goals/plans? |
|  | 1. How do you feel this internship has helped you to achieve your goals? |
|  | 1. Would you take part in another supported internship programme? |
|  | 1. Which one thing could have been better about the internship? |
|  | 1. What was the most useful thing you learnt from the internship? |
|  |  |

1. **Post-internship Manager Interview Schedule**

|  |  |
| --- | --- |
| **Primary question** | **Probe questions** |
| Tell me a bit about how the internship programme for [autistic] graduates went over the past 3 months? | 1. What benefits/opportunities do you think Deutsche Bank – or the individual teams – gained as an organisation? |
|  | 1. How have the people working at Deutsche Bank benefited from the programme? |
|  | 1. What do you think that the autistic interns got out of the programme? |
|  | 1. How did they get on in terms of: |
|  | 1. the quality of their work |
|  | 1. the social side of work |
|  | 1. practical things (e.g., getting to work, dress code etc.) |
|  | 1. Would you consider the internship programme a success? What is your main indicator of this ‘success’? |
| What about challenges that you encountered during the programme, if any? | 1. What were the things that were most challenging for you and your non-autistic colleagues with respect to the interns/internships? |
|  | 1. What do you think were some of things that were most challenging for the autistic interns? |
|  | 1. How much support did they need? In what areas did they need support? |
|  | 1. What accommodations, either personally or in the workplace, did you make to accommodate the autistic interns? |
| [for managers of autistic interns] Can you tell me about the training you received ahead of the internship? | 1. Did you attend the training on autism offered by Deutsche Bank (1 hour briefing or 3 hour training)? |
|  | 1. In what ways was the training helpful? |
|  | 1. Is there additional training that you think could have been helpful? |
| Let’s talk a bit about the outcomes of the internship for you | 1. What was the most useful thing you learnt from the internship? |
|  | 1. What would you do differently next time if you could make changes? |
|  | 1. What advice would you offer to other firms considering running a similar programme? |
|  | 1. Would you be involved in another supported internship programme? |
