# Supplementary material for: Experiences of autistic and non-autistic individuals participating in a corporate internship scheme
Source: Autism. 2021 Jun 19;26(1):201–16. doi: 10.1177/13623613211025115 (PMC8750129; doi:10.1177/13623613211025115)
Supplement: sj-docx-2-aut-10.1177_1049732320931430 – Supplemental material for Experiences of autistic and non-autistic individuals participating in a corporate internship scheme [file sj-docx-2-aut-10.1177_1049732320931430.docx]

**Supplementary Materials B. Intern and manager Work Performance Questionnaire scores at the conclusion of the internship**

| **Variable** | **Autistic intern** | **Non-autistic intern** |  |  | **Manager of autistic intern** | **Manager of non-autistic intern** |  |  |
| --- | --- | --- | --- | --- | --- | --- | --- | --- |
| **Work Performance Questionnaire**  **Frequency** | **Mean (SD)**  **n = 12** | **Mean (SD)**  **n = 8** | **Sig.** | ***r_equiv._*** | **Mean (SD)**  **n = 7** | **Mean (SD)**  **n = 4** | **Sig.** | ***r_equiv._*** |
| Presentation, punctuality, & responsibility | 4.6 (0.3) | 4.9 (0.1) | .016 | -0.55 | 4.8 (0.2) | 5.0 (0.1) | .527 | -0.23 |
| Task comprehension and planning | 4.2 (0.6) | 4.9 (0.3) | .004* | -0.63 | 3.5 (0.7) | 4.8 (0.3) | .006* | -0.77 |
| Task performance | 4.3 (0.6) | 4.8 (0.3) | .069 | -0.42 | 3.9 (0.5) | 4.6 (0.3) | .012 | -0.75 |
| Dealing with distractions | 4.1 (0.6) | 4.9 (0.2) | .001* | -0.69 | 4.0 (0.7) | 4.9 (0.1) | .024 | -0.67 |
| Contact/interaction with colleagues and superiors | 4.4 (0.6) | 4.9 (0.1) | .025 | -0.51 | 4.5 (0.3) | 4.8 (0.2) | .164 | -0.46 |
| **Work Performance Questionnaire**  **Independence** | **Mean (SD)**  **n = 12** | **Mean (SD)**  **n = 8** | **Sig.** | ***r_equiv._*** | **Mean (SD)**  **n = 7** | **Mean (SD)**  **n = 4** | **Sig.** | ***r_equiv._*** |
| Presentation, punctuality, & responsibility | 4.8 (0.3) | 5.0 (0.0) | .012 | -0.63 | 5.0 (0.0) | 5.0 (0.1) | .527 | 0.40 |
| Task comprehension and planning | 4.4 (0.8) | 5.0 (0.1) | .020 | -0.56 | 3.5 (0.8) | 4.8 (0.3) | .012 | -0.72 |
| Task performance | 4.5 (0.5) | 4.9 (0.2) | .031 | -0.51 | 4.0 (0.5) | 4.7 (0.4) | .024 | -0.66 |
| Dealing with distractions | 4.5 (0.9) | 5.0 (0.0) | .069 | -0.51 | 4.3 (0.6) | 5.0 (0.0) | .006* | -0.82 |
| Contact/interaction with colleagues and superiors | 4.6 (0.5) | 5.0 (0.1) | .016 | -0.57 | 4.6 (0.3) | 4.8 (0.2) | .109 | -0.53 |
