# Supplementary material for: Experiences of autistic and non-autistic individuals participating in a corporate internship scheme
Source: Autism. 2021 Jun 19;26(1):201–16. doi: 10.1177/13623613211025115 (PMC8750129; doi:10.1177/13623613211025115)
Supplement: sj-docx-3-aut-10.1177_1049732320931430 – Supplemental material for Experiences of autistic and non-autistic individuals participating in a corporate internship scheme [file sj-docx-3-aut-10.1177_1049732320931430.docx]

**Supplementary Materials C. Themes and subthemes, with example quotes for interns’ and managers’ experiences through the internship.**

1. **Intern Themes**

|  |  |  |
| --- | --- | --- |
| **Themes** | **Sub-themes** | **Illustrative Quotes** |
| 1. A journey from apprehension to self-assurance | *1.1 Excitement and nervousness* | AI: “I’m quite excited. I’m also a bit nervous because I’ve never worked in... it’s quite a kind of intense environment.” [A-I3] |
|  |  | NAI: “I’m actually really excited. I’m obviously a bit nervous as well, just it’s more the fear of the unknown, I’d say, and just don’t know exactly what’s expected, how much they presume I will already know and things. But hopefully I can just ask if I’m stuck on anything and it’ll be fine so...” [NA-I14] |
|  |  |  |
|  | *1.2 Negative past work experiences** | AI: “Basically, I got about two or three assessment centres but I was rejected after the assessment centre. Mainly the feedback was that I didn’t... so interaction skills, interpersonal skills, and I wasn’t very enthusiastic for – that’s what they said – for the assessment centres.” [A-I7] |
|  | *1.3 Hopes for success and development through the internship* | AI: “It’s the fact that I’ll be doing something completely different to what I’ve been doing on a day to day basis. And hopefully I’ll find it, I’ll be given some challenging work to really kind of get my teeth into and some good problem solving. Because at the moment in my previous job I never really … I never really felt challenged, so I’d really like to be challenged.”[A-I4] |
|  |  | NAI: “Definitely looking forward to some, like, sense of that you’ve done something. So like, the sense that you’ve completed a task and it’s going to actually have an impact on your team. I think like, that, I’m really looking forward to actually doing meaningful work to the team.” [NA-I4] |
|  | *1.4 Boost in confidence and self-esteem* | AI: “I think one of the key things about the whole thing was that I was able to use my initiative from time to time, always thinking about how to do something better and I think she admired that from me.”[A-I12] |
|  |  | NAI: “I’ve gained confidence in my abilities, which I think was previously probably lacking a bit.” [NA-I1] |
|  |  |  |
|  | *1.5 Improved technical and tacit skills* | AI: “I mean, for three months, I’ve managed to gain experience which is absolutely priceless and I gained, I’ve not only felt like I’ve further improved on skills I’ve gained before joining this internship, but I feel like I’ve gained lots of new and different skills I could’ve never thought I would have achieved. It’s been absolutely great.” [A-I8] |
|  |  | NAI: “We got some really excellent training on presentation skills in the internship, which was useful.” [NA-I1] |
|  | *1.6 Raised ambitions for the future* | AI: “My goals and plan is certainly to stay in DB for as long as possible, because DB has opened many doors for me they can’t possibly imagine so...” [A-I8] |
|  |  | NAI: “Yeah, no, I think it’s helped me so much because the experiences that I’ve kind of got from this internship and the responsibilities that I’ve had—because I’m just applying for part-time jobs now for next year—will help me so much, and I suppose if you say you’ve worked for an investment bank it’s quite impressive.” [NA-I11] |
|  |  |  |
| 2. Perceived contributors to success | *2.1 Highly motivated to participate in internship* | AI: “I think it’s going to look great on my CV, that sounds cynical, three months Deutsche Bank is probably going to look pretty good, and the…just learning new skills, being able to show that I can do those skills on my, yeah for future employers and, who knows, if this goes well, who knows.” [A-I10] |
|  |  | NAI: “So that kind of thing, and I know that Deutsche Bank are huge and they’re global...and the kind of stuff I’ll be working on will really impact people.”[NA-14] |
|  | *2.2 A more welcoming recruitment process* | AI: “When I came in for both my interviews, both of them were really, really nice. Very accommodating. They were very friendly and it felt like a very relaxed environment, it made it seem like an informal meeting.” [A-I11]  NAI: “Oh it was really nice. I thought it was one of the nicest ones I did actually. Like there wasn’t too many long-winded questions online... and then the phone interview was lovely, And then the assessment centre, both the interviewers I had were really, really nice as well in that.” [NA-I14] |
|  |  | NAI: “So that kind of thing, and I know that Deutsche Bank are huge and they’re global...and the kind of stuff I’ll be working on will really impact people.”[NA-14] |
|  | *2.3 An autism-friendly workplace** | “Basically because Deutsche Bank, I really like this company because I find that even though it’s making a profit right now, it was making losses before, and I think it was really friendly to autistic people and not many other companies are as friendly to autistics as Deutsche Bank are.” [A-I13] |
|  | *2.4 Ability to rely on a strong support network* | AI: [My family] have been really supportive. They’ve understood any kind of anxiety or problems that I might face prior to it and they’ve spoken to me about anything that could possibly be going through my mind.” [A-I4]  NAI: “I have a good relationship with my parents, so if something comes up I’ll speak to them. I have a group of friends, I have other friends actually who are doing similar internships so, they may have experienced something similar as well so it may be good to speak to them. Yeah, I feel like I’m good in terms of people to talk to.” [NA-I9] |
|  | *2.5 Insight into own strengths and challenges** | “I’m organised. I’m a quick learner. I’m very honest, and I’m straight to the point” [A-16].  “My main weakness would be, I would say, my interpersonal skills in terms of how I am perceived by others...So from my experience these are my main issues. ” [A-I2]  I think my weaknesses would lie when I’m not exactly sure what I’ve been tasked to do. So, if I’ve been told to just, “Here is some information, analyse this,” without showing which area they want me to analyse, what the trends they kinds of want to find, it’s quite difficult.[A-I5] |
| 3. A challenging process | *3.1 Lack of clarity and organisation* | AI: “I guess one thing which a number of the interns have talked about is...a lot of things have been not that well-organised. So, they’re things like last minute changes.” [A-I1] |
|  |  | NAI: “So my mentor, she wasn’t aware that she had multiple mentees so for about four weeks she didn’t respond to two of these.” [NA-I7] |
|  |  |  |
|  | *3.2 Feeling ‘othered’ by the work environment** | AI: “I think it has got me thinking about my autism for the first time in possibly nearly ten years, to any great detail...no in about five or six years, since I was beginning to look at university...And, even then it wasn’t that great. This is the most I’ve sort of had to think about it since early high school.” [A-I10] |
|  | *3.3 Misunderstandings arising from communication differences** | AI: “Weaknesses yeah, I would say mostly my communication skills because I tend to isolate myself in a task...I mostly prefer to just be alone and work on a task...I cannot follow when there’s a meeting and, it’s a point of, you know, you have different messages? I cannot process them very fast.”[A-I7]  “…it really put my head in a downward spiral, and then a couple of days I had a word with him about it and he said, ‘ah, that was my fault, I shouldn’t have worded it the way I did”. [A-I8] |
|  | *3.4 Reluctance to ask for help or supports** | AI: “Sometimes I do struggle with asking for help because I kind of feel a bit stupid having to ask sometimes.” [A-I4] |
|  | *3.5 Mental health struggles** | AI: “Generally, the most apparent thing when I start new things is anxiety so I get quite anxious dealing with the unknown, it causes me anxiety.” [A-I7] |
|  |  |  |
| 4. The path to better outcomes | *4.1 Consistency in support and communication* | AI: “I would guess just organisation and communication probably. So, making sure that the information that was given to us was consistent.” [A-I5] |
|  |  | NAI: “They could have just communicated better and made sure that the way the process goes through there are no situations like that that happen. But it happened all the time.” [NA-I1] |
|  | *4.2 More networking opportunities for interns* | AI: “I think it’s just mainly to do with how we were trying to network and stuff. Because a lot of the interns didn’t know how to network properly and they’re going in, blindfolded, going in and saying, you know, ‘Hey can I please get a job?’ And there’s a particular way how to treat people and stuff. [A-I11]  NAI: “I really would have liked the opportunity to kind of spend a day shadowing a completely different division just to kind of broaden out that network again, because that was something that was really emphasised throughout and I thought it was a bit limited to maybe reduced networking just to like emailing someone and being like, ‘Oh can you go for a coffee with me please?’” [NA-I12] |
|  | *4.3 Managers and co-workers need better intern-specific knowledge** | AI: “The training I think my colleagues received was not tailored to me which I think is a problem, because how can you deliver training about autism, is a spectrum and it’s a very, very wide spectrum and among the interns that there are on my scheme there is a range of people at this high functioning end of the spectrum, people who struggle a lot more socially than others.” [A-I3] |
|  |  |  |

* Theme specific to autistic interns

1. **Manager Themes**

|  |  |  |
| --- | --- | --- |
| **Themes** | **Sub-themes** | **Illustrative Quotes** |
|  |  |  |
| 5. An opportunity with mutual benefits | *5.1 Boost team productivity* | AI: “And particularly around certain things that we do that, you know, I think potentially could lend themselves well to people with some capabilities on the spectrum of autism.” [A-HM4] |
|  |  | NAI: “taking on an intern is actually a good way of actually getting eight weeks’ worth of work done” [NA-HM2] |
|  | *5.2 Maximise interns’ skills, CV and confidence* | AI: “I think they had the opportunity to obviously work closely with the team, get some exposure with different people, understand how different aspects within the organisation works, so it was quite a rich experience for them.” [A-HM6] |
|  |  | NAI: “during the midterm feedback cycle that me and [co-worker] gave her [intern], we were saying, “Look, don’t be afraid to speak your mind, really ask us questions, if nothing makes sense then just call it out.” She really took that onboard and in the second half of the internship, you could definitely tell that she was sort of picking up the phone more and really asking questions of anyone in the team” [NA-HM1] |
|  | *5.3 Enriching the organization as a whole* | AI: “I think we’re missing out on a whole set of talented people that we wouldn’t normally get, I mean a bit of a generalist statement but I think that, yeah, particularly with technology given the type of work that we’re doing and the struggles that we have to get really good, you know, technically competent people, you know, it’s actually quite hard to find people in London, we’re missing out on a whole market of people.” [A-HM9] |
|  |  | NAI: “And I think also, from my perspective and Deutsche Bank’s perspective, I think one of the main aims for us is also to get some insight into the talent and also maybe get some insight into some new ideas and how we need to be moving forward and how we should be thinking about the people we’re bringing onboard.” [NA-HM2] |
|  | *5.4 New management practices** | AI: “I think we could learn a lot from, you know, people that sort of are different to us, you know, broaden the sort of managerial skills and for the interpersonal skills and […] broaden your […] exposure to people which, you know, could probably sometimes be quite narrow in the normal day to day that you get in a working environment like this” [A-HM1]  AI: “I think it was a positive experience. I think we’re often, often guilty of operating in the way that, that WE operate and not making any allowance for other people at all and this really forced us to think about that side of things and you know that can have wider benefits in our engagement across the teams, particularly when we are dealing with some of the different global teams, and there are different challenges and different ways of working and better ways of consuming information and sharing information, so I think that was positive.” [A-HM10] |
|  | *5.5 Improve brand reputation** | NAI: “Again I think it's positive for Deutsche, from a perspective outside looking in, to be supporting this type of thing. I think Deutsche and I think a lot of the financial industry accepts they've fundamentally got to change they the way operate. Things like the financial crisis have changed things to a reasonable extent, but there's still a long way to go from all sorts of equal opportunities side of things, from the type of people we are working with.” [A-HM10] |
|  |  |  |
|  |  |  |
| 6. The key to success | *6.1 Integrate into the team* | AI: “Hopefully that individual is able to grasp the concepts of virtually working in a global team which is how we operate and how a lot of the support teams operate.” [A-HM8]  AI: “He integrated with the team, integrated with the department.” [A-HM2] |
|  |  |  |
|  |  | NAI: “I guess I would be disappointed if they just sort of sat at their computer and acknowledge everyone else, and didn’t socialise. We’re not a group of individuals, we’re a team.” [NA-HM3] |
|  | *6.2 Matching intern skills to job role* | AI: “I think the recruitment is a key thing here and recognising the background, the skillset of the candidates and trying to match it to the right thing. It’s like for anybody else, if you place someone in the team that is not suitable for a candidate then it’s not going to be a success or unlikely to be a success.” [A-HM8] |
|  |  | NAI: “they weren’t a great fit, it’s basically because they didn’t have a bunch of technical skills that are mandatory for our group and the quality of the work reflected that” [NA-HM3] |
|  | *6.3 Importance of interns taking initiative* | AI: “Typically as well, a lot of the team are fairly I guess self-sufficient and although I'm managing them, it's relatively hands-off and there's not necessarily for a lot of the roles a very, very specific process that they go through every day” [A-HM10] |
|  |  | NAI: “I think what I am trying to say is, people have to understand that if there’s a deliverable due, and if you’ve got your work on, you need to look at your work, prioritise what’s more important, and focus on that to get it done in time, and really, it’s down to the individual to work that out.” [NA-HM4] |
|  |  |  |
|  | *6.4 Meaningful, appropriate work* | AI: “I think the only thing I am concerned about is making sure that I have enough for him to do, because it is quite difficult to package things up isn’t it, for a short period of time?” [A-HM11] |
|  |  | NAI: “I guess I have apprehensions for me and my colleagues in that we’re going to get an intern who actually is effectively over-challenged by our work environment.” [NA-HM3] |
|  | *6.5 Effective communication** | AI: It was hard sort of to communicate verbally and exactly find out whether I was being understood or not, or whether what I was... It was hard to say. You know, I was giving him a [a task] and I couldn’t tell whether I was making total sense when I was like explaining something that should be as obvious as that wall over there, or whether he wasn’t getting one word I was saying at all. So I had to definitely adapt that style to say […] “Does that sound easy, medium or hard?” He always said medium. I didn’t know exactly, yeah, whether he’d got it or not.” [A-HM1] |
|  |  | AI: “I think when you're looking at communication, that brings its own challenges within often a fairly pressurised environment. I think sometimes where there's things in terms of processes or ability to handle certain dialogue, obviously that brings some challenges, but again very much depends on the individual.” [A-HM10] |
| 7. Individualized support | *7.1 Initial concerns about not knowing when or how to provide support** | AI: “No, I think it’s that and then to be honest probably the biggest thing is being able to get the right balance between being able to spend time without mothering them, if that makes sense, you know, because obviously you’re used to spending time with, again one to ones and stuff we don’t tend to do too formally here it tends to be as it’s needed, obviously you’re setting up one to ones but it’s like how frequently do we do it? Do we do it every day? Do we do it every two days?” [A-HM9] |
|  |  |  |
|  | *7.2 Facilitated key adjustments for the intern** | AI: “the way that I conveyed information and the task requirements, I was more specific, less ambiguous probably than I would have been sometimes.” [A-HM4] |
|  |  | AI: “so there was flexibility with regards to her working hours. She left at 4:30, whereas obviously a lot of our office work later. She got in earlier and she took a lunch break. These are things that we don’t generally tend to do. I haven’t had a lunch break myself for years. It’s something that was understood that was part of how she worked, and that was accommodated.” [A-HM7] |
|  | *7.3 Level of adjustment and investment in support varied greatly** | AI: I think the main adjustment is to recognise the individual, what their needs are, and to be proactive at observing, and obviously tailoring, and flexing, and being agile, so we’re sure that we’re able to provide that.” [A-HM7] |
|  | *7.4 Poised to make adjustments on the fly** | AI: “He was sitting next to someone one day and he asked me if I could hear this buzzing sound (laughs) and it turned out it was from... the guy next to us charged his mobile phone in this particular socket, not any other one but this particular one, it sort of made this high frequency buzz which he could hear and so on. (Laughing) Yeah, and we sort of asked him to just put it into the next socket and that was fine” [A-HM1] |
|  |  |  |
| * Theme specific to autistic interns | | |
